# The Case for a European Credit Council: Historical and Constitutional Fine-Tuning

**DOI:** 10.1515/ael-2022-0074

**Published:** 2023-01-03

**Authors:** Jens van ’t Klooster

**Affiliations:** Department of Political Science, University of Amsterdam, Nieuwe Achtergracht 166, Amsterdam, Netherlands

**Keywords:** European Credit Council, Central Bank independence, democracy, credit policies, sustainable finance, E52 – Monetary Policy, ME58 – Central Banks and Their Policies, N24 – Europe: 1913–

## Abstract

Eric Monnet’s European Credit Council (ECC) is an innovative, historically-grounded institutional proposal for supporting the ECB in the design of its monetary policy operations. In this commentary, I seek to strengthen the case for the European Credit Council drawing on work in progress on the history of the ECB. I first discuss the tradition of moderate interventionism as it appears in Monnet’s (Monnet, E. (2018). *Controlling credit: Central banking and the planned economy in Postwar France*, *1948–1973*. Cambridge University Press) study *Controlling Credit*. I show that the model of moderate interventionism was well-known to the drafters of the ECB statutes and efforts to categorically rule such policies out were simply unsuccessful. I suggest that this fortuitous choice has left ample legal space in the EU treaties for an ECC.

## Table of Contents

1 Market-Based and Moderate Interventionist Central Banking in the Early 70s
2 The ECB’s Turn to Moderate Interventionism
3 A Credit Council Today
4 Conclusion
References

## Toward a European Credit Council?

1. The Democratic Challenge of Central Bank Credit Policies, by Eric Monnet, https://doi.org/10.1515/ael-2022-0113.
2. The Changing and Growing Roles of Independent Central Banks Now Do Require a Reconsideration of Their Mandate, by Charles Goodhart and Rosa Lastra, https://doi.org/10.1515/ael-2022-0097.
3. Is Asking Questions Free of Charge? Questioning the Value of Independent Central Banks through the Lens of a European Credit Council, by Matthias Thiemann, https://doi.org/10.1515/ael-2022-0108.
4. The Democratic Dangers of Central Bank Planning, by Nathan Coombs, https://doi.org/10.1515/ael-2022-0063.
5. A European Credit Council for Consistent and Informed Policymaking, by Agnieszka Smoleńska, https://doi.org/10.1515/ael-2022-0065.
6. The Case for a European Credit Council: Historical and Constitutional Fine-Tuning, by Jens van ’t Klooster, https://doi.org/10.1515/ael-2022-0074.
7. A European Credit Council? Lessons from the History of Italian Central Banking after World War II, by Mattia Lupi, https://doi.org/10.1515/ael-2022-0071.
8. The Power of Coordination and Deliberation, by Eric Monnet, https://doi.org/10.1515/ael-2022-0114.
9. The Credit Council in the US Context, by John Davis Feldmann, https://doi.org/10.1515/ael-2023-0134.
10. Democratic Central Banking: Power Not Deliberation, by Leah Rose Ely Downey, https://doi.org/10.1515/ael-2022-0073.

Eric Monnet’s (2021a, 2021b, 2022 European Credit Council (ECC) is an innovative, historically-grounded institutional proposal for supporting the ECB in the design of its monetary policy operations. The ECC would be a new European institution, which could constitutionally fall under the European Parliament, and have two roles. First, to improve the quality of deliberation on European monetary policy by putting forward an independent assessment of decisions, in particular where these have important distributional effects or are in other ways significant. I imagine a body that pursues investigations such as the House of Lords’s review of the Bank of England’s quantitative easing policies, but has the investigatory powers of an independent assessment office. That strikes me as an important improvement over the EP’s current accountability practices, but I will not say more about it here.1The outcome of the review can be found in House of Lords (2021). On improving EP-ECB accountability through ex post reviews and an independent evaluation office, see van ’t Klooster and Grünewald (2022). Instead, I want to focus on the ECC’s role in developing proposals for designing monetary policy. Alongside price stability, its legal mandate instructs the ECB to support the general economic policies in and of the European Union. As a forum for inter-institutional coordination, the ECC would be empowered to make proposals for the design of ECB monetary policy operations in line with the ECB’s secondary mandate.

**Table 1:** EEC central banks, governance structure and allocative policies in 1972.

	Market-based (DE, NL)	Moderate interventionism (BE, FR, IT)
Control of credit provision	Domestic monetary policy steers the overall volume of credit but not its allocation	Domestic monetary policy steers the overall volume of credit while making some allocative choices
Governance	Independent agency	Inter-institutional coordination

In the following, I seek to strengthen the case for the European Credit Council. I first discuss Monnet’s (2018) study *Controlling Credit*: *Central Banking and the Planned Economy in Postwar France, 1948–1973* to identify a tradition that I describe as “moderate interventionism”. I contrast the Banque de France and its relationships to the French National Credit Council as described by Monnet with the more market-based monetary policy of Germany and the Netherlands around the same time. I then turn to the creation of the EMU to show that the model of moderate interventionism was well-known to the drafters of the ECB statutes and efforts to categorically rule such policies out were simply unsuccessful. As a consequence, there always remained ample room for an interventionist central bank in the ECB statutes, space that the central bank has made considerable use of in the past years. I conclude by outlining the legal space available within the European Treaties for Monnet’s proposed European Credit Council, which in light of historical experience seems a welcome complement to the new role that the ECB is taking up today.

## 1 Market-Based and Moderate Interventionist Central Banking in the Early 70s

In the early seventies, as Monnet shows in his magisterial *Controlling Credit*, the Banque de France was in important regards distinct from the types of central banks we know today. If we look at the central banks of the European Economic Community during the early 1970s, we see two distinct models: one market-based, the other moderately interventionist (Table 1).

The Dutch and German central banks sought to steer the overall volume of money creation but largely stayed out of the allocation of credit by banks. In Belgium, France and Italy, in contrast, the central bank was embedded in a broader public framework for the allocation of credit. As a policy report from the period explained (with a lot of carefully drafted formulations), disagreement revolves around the proper bounds between the central bank and the market:

For reasons of tradition – a reflection of which is found in the institutional framework and in the distribution of powers among the monetary authorities – the national authorities attach to the principles that inspire their action a weight that is not the same for all the countries concerned. In some of them preference is given to the market mechanisms; in others, on the other hand, more importance may be attached to direct intervention that imposes more or less constraint on the authorities. (EEC, 1972, p. 44)

The German Bundesbank resembled in some ways (though not in others) what central banking would come to look like in the 1980s. The central bank set the interest rates for its discount facilities and open market operations to stave off domestic inflation. In this context, it operated as a largely independent institution, which left the allocation of credit to a complex web of private, semi-private and public lenders. Alongside the Bundesbank, public allocation of credit occurred on a large scale by development banks, in particular the *Kredit für Wideraufbau* (KfW) and publicly owned regional banks. So, although the allocation of credit in the German economy was far from purely market-based, the central bank’s role in economic policy would be narrow, allowing it to operate largely in isolation from the treasury. The Dutch central bank, as the *Bundesbank*’s little brother, was only slightly less independent and equally hesitant to intervene in the allocation of credit (Den Dunnen, 1973; EEC, 1972, pp. 275–347).

The Belgian, French and Italian central banks were much more closely integrated into the state (EEC, 1972; Monnet, 2018, Chapter 7). These central banks set their policies in close coordination with treasuries, economic planning boards and specialized credit councils. Decisions were made with attention to the broader economic policies which the financial system was meant to achieve. Initially set up to support the recovery efforts, this institutional structure would remain a crucial pillar of domestic economic policy. During the 1960s and early 1970s, a turn to an increased role for the market and more price-based instruments had gone together with a continued policy of targeted allocation of credit.

In France, responsibility for monetary policy was shared between the central bank and a National Credit Council (*Conseil National du Credit*), which held a “consultative and initiatory role in everything concerning money and credit” (EEC, 1972). Despite considerable flaws from the perspective of today’s standards of input and throughput legitimacy, they provided an additional forum for deliberation on the general orientation of financial governance (Monnet, 2021b). Alongside the Treasury and the central bank, the Council consisted of “a further 45 members representing Government Departments, banking and financial bodies, users of credit and trade union organizations”, as well as the Principality of Monaco (“for any particular discussions of decisions of concern to it”) (EEC, 1972, p. 137).

Together, the two institutions set monetary policy as part of France’s broader architecture of economic planning. Since interest rate policy was used to stabilize the exchange rate, domestic policy rested primarily on three instruments (Monnet, 2018, pp. 173–7, 140–9). First, the central bank used quantitative limits on refinancing credit, but this instrument became less important from 1958 onwards and was finally abolished in 1972. Second, traditional reserve requirements went together with liquid asset ratios, which required banks to hold a minimum ratio of medium-term credit. Finally, the central bank used a system of credit ceilings (*encadrement de credit*) to slow down bank lending in 1958–9, 1963–65 and 1968–70. During the 1970s, the volume of credit exempt from the ceiling would go up from 8% in 1972 to 18.5% in 1975 (Monnet, 2018, p. 124).

However, as Monnet also makes clear, the differences between the models shouldn’t be exaggerated. In none of the 1972 EEC members was the central bank the main agent in allocating credit. Although the Banque de France had an important supporting role, its primary tasks remained quite similar to those of today’s central banks, seeking to steer the overall volume of money while keeping an eye on the business cycle and the balance of payment. As Monnet explains,

The archives of the Banque de France and *Conseil National du Crédit* […] show little evidence of compulsory guidelines that forced the banks to lend (or prevented them from lending) to specific firms or sectors. […] The few examples available in the archives of the CNC are related to agricultural credit and were intended to avoid overproduction. For example, the Ministry of Agriculture decided to control loans to the chicken farming business because it was concerned with overproduction and big inventories. In July 1961, it asked the Banque de France and the CNC to prevent banks from lending to businesses that raised more than 5,000 chickens. The limit was then extended to 16,000 chickens in 1963. However, this “chicken example” is not representative of the central bank’s interventions in the allocation of credit throughout the *dirigiste* period. (Monnet, 2018, p. 226)

The Banque de France’s allocative choices were used to fine-tune the rules or ward-off undesirable side effects. Consider the exemptions from the 1968 *encadrement* (BIS, 1971, pp. 31–33)*.* For one, higher ceilings applied to banks with a seasonal business model, financing inventory stock or the tourism sector. The largest exemptions were for sugar crop producers, which had been at a very low level of credit when the ceiling was imposed in October. Second, ceilings were relaxed for new banks, banks with new branches and those that had just taken large commitments. Third, some economic policy choices were made in exempting short-term credit for the stockpiling of cereals and meat production (“which needed to be encouraged”). Fourth, the central bank exempted “loans for the resettlement of repatriated French citizens, loans for the construction of farm buildings subsidized by the Ministry of Agriculture and loans for farmers suffering losses as a result of national disasters”. Fifth, additional facilities were provided to banks focused on financing export. Large banks were not eligible for any of these exemptions as they would be able to “strike a balance between the needs of their numerous customers and of the different economic sectors and regions”. While the wisdom of any of these individual exemptions could of course be debated, it takes considerable ideological rigour to find much to reject here as a matter of principle.

## 2 The ECB’s Turn to Moderate Interventionism

Against the historical background of Monnet’s *Controlling Credit* we can see that the European Central Bank has recently moved away from an entirely market-based approach towards a model of central banking closer to the 1970s moderate interventionist model.2This section and the next incorporate ideas on the ECB’s legal framework developed in De Boer and Van ’t Klooster (2020), Van ’t Klooster and de Boer (2022) and van ’t Klooster and Grünewald (2022). This is a turn for which the drafters of the ECB statutes had always left ample scope, as a Dutch central banker involved in the drafting noted (with regret):

For France, in particular, accepting the Bundesbank model was a major concession, deviating as it did from French centralist tradition, and even raising doubts on the purpose of monetary integration, which for France was to ensure more influence on monetary decision-making. Thus having in principle made the concession at the European Council Meeting in Rome in October 1990, it fought a rearguard action when the European Central Bank’s statutes were drafted. Though the principle of independence of the ECB and its priority for price stability was clearly established in the Treaty, sufficient ambiguity was incorporated in it to make sure that the issue could be raised again at a later stage. (Szász, 1999, p. 147)

The early ECB emerged from the interpretation of terse passages in the Maastricht Treaty put forward by the European Monetary Institute (1994–8) and its first board members. In the early strategies, the ECB assigned itself a narrow role in using refinancing conditions for banks to steer interest rates in interbank markets (ECB, 1998, 2003). Following this self-understanding, the democratic legitimacy of the central bank rested on using only very limited discretion in the exercise of its task. The ECB would pursue one objective; “price stability” as spelt out in Article 127(1) Treaty on the Functioning of the European Union (TFEU). The ECB’s role as a monetary policymaker was taken to consist primarily in finding the level of interest rates compatible with medium-term price stability. Although the term itself was not used by the ECB to refer to its own operations, these were in practice broadly what the central bank today refers to as market neutral (Bindseil & Papadia, 2006; cf. Cheun et al., 2009). It was left to banks to allocate money over the European economy subject to the price incentives of the ECB’s money market interventions.

These early practices of interpretation reflect a specific set of ideas prevalent in the 1990s and 2000s. However, little of these ideas made it into the legal text itself. For example, the choice to observe a strict principle of market neutrality always had only a shallow basis in the text of Article 127(1) TFEU. Other provisions favour more interventionism. In setting monetary policy, the Treaties explain, the ECB does not only “implement”, but also “define” its monetary policy (127(2) TFEU). At the same time, the ECB does not only have an objective of price stability, but also a secondary mandate which requires that “without prejudice to the objective of price stability, the [ECB] shall support the general economic policies in the Union”. The provision continues to specify that the ECB should support those general policies to contribute to the objective of the EU as outlined in Article 3 of the Treaty on European Union. An equally indeterminate provision holds that the ECB should act “in accordance with the principle of an open market economy” (127(1) TFEU; Treaty on the Functioning of the European Union). As the commentary to the provision explains, it is primarily meant to end the use of directly allocative instruments such as credit ceilings:

this Article enables the ECB and national central banks to regulate indirectly – and without recourse to administrative controls or restrictions – money and credit market conditions. This form of monetary management relies on financial incentives, leaving it to private market participants to respond voluntarily [.] (CoG, 1990a, p. 16)

As the Dutch central bank governor Wim Duisenberg already observed while drafting the provision, it did not obviously make much sense:

some of the actions undertaken by central banks could always be regarded as inconsistent with free and competitive markets; for example, the setting of key official interest rates could be seen as an exogenous act which might not be in conformity with local market conditions. (CoG, 1990b, p. 4)

His French colleague Jacques de Larosière added that the central bankers “should be careful not to limit the scope of the System”, which “should be evolutionary and designed to deal with unforeseen circumstances” (CoG, 1990b, p. 4). The commentary later published with the Statutes contains almost those exact words; the operational provisions were drafted “with due regard to the evolutionary nature of financial markets” thereby seeking to ensure that the central bank could “respond adequately to changing market conditions” (CoG, 1990a, p. 16).

If the governors were ever bent on providing the ECB with strict instructions, it is hard to see much fruit from their labour in the mandate as drafted. In line with the French push for flexibility, there is almost nothing in the Statutes concerning how the ECB should pursue its objectives. Article 18 of its Statute permits the ECB to engage in any financial market transactions required. In preparatory notes, central bankers point out that it allows for “rediscounting at a preferential rate with ceilings by bank or for certain types of credit” subject to the caveat that the ESCB would “follow the same rules in all the member states” (CoG, 1990c, p. 19). The Governor of the Central Bank of Ireland still foresaw a risk of locking the central bank into “a prescribed method of monetary control”, so that the current Article 20 was subsequently added. This provision permits the ECB “the use of such other operational methods of monetary control as it sees fit”, conditional on a two-thirds majority in the Governing Council. Article 123 TFEU prohibits the direct purchase of public debt, but (famously) allows for “the purchase of government bonds in securities markets” (CoG, 1990a, p. 11; cf. Orphal et al., 2022); which economically amounts to almost the same thing.

In the past years, the ECB has moved decisively beyond the market-based model of central banking, once again embracing a form of moderate interventionism. Most changes in its operations predate the 2021 Strategy, but the new monetary policy framework does a lot to codify and formalize a more interventionist role. While I lack the space here to explore the legal and political justification of these changes, it is clear that by now a striking asymmetry has emerged. The ECB has used the space available within its mandate to take on a much more political role but left its accountability practices almost unchanged. It is in light of this asymmetry that a European Credit Council is needed for dealing with the ECB’s new-found role of supporting the EU’s general economic policies.

Although for a long time absent from its public statements, the ECB has always pursued objectives other than price stability through the design of its monetary policy operations. Managing risk on its refinancing operations, for example, has the objective of protecting the Eurosystem against potential losses due to counterparty default. After the 2008 Lehman Brothers crash, the ECB started using the design of its monetary policy operations to promote transparent securitization practices in support of financial stability. At the time of the default, Lehman Brothers Bankhaus AG, a Frankfurt-based German subsidiary, had a debt of €8.5 billion to the German Bundesbank (Deutsche Bundesbank, 2015; Euromoney, 2009). To secure this debt, Lehman Brothers had pledged 33 securities – primarily “highly complex” asset-backed securities (ABSs) with exotic names such as “Diversity”, “Excalibur”, and “Ruby”. In the years that followed, the ECB used eligibility requirements on collateral to enforce more rigid standards on asset-backed securities (Braun & Hübner, 2018). Similarly, the inclusion of asset-backed securities, covered bonds and, in particular, corporate bonds in ECB QE also served to promote the EU’s project of a capital markets union. More controversially, the ECB also took up a much more selective role in intervening in sovereign debt markets during the 2010–12 Eurozone crisis; its security markets programme (SMP) involved purchases roughly similar in scale to those of the Federal Reserve’s purchase of treasuries in its QE operations.

The 2021 strategy review resulted in important revisions to the ECB strategy (van ’t Klooster & Grünewald, 2022). Alongside a symmetric 2% objective for the growth of consumer prices on a medium-term time horizon, the central bank now takes into account a broad range of economic preconditions of price stability. Most prominently, the ECB announced that it would take a more active role in monitoring the effects of its policy on climate and the environment. This followed several years in which the European Parliament and NGOs raised concerns based on academic research linking the ECB’s corporate bond purchases to the most polluting sectors of the European economy (Cojoianu et al., 2020; Dafermos et al., 2020; Matikainen et al., 2017). On the basis of the new strategy, the ECB has set out to revise its operations with an eye to the EU’s climate objectives (ECB, 2021).

In its July 2022 announcements of operational changes, the ECB no longer explicitly identifies price stability as the objective of specific greening measures (ECB, 2022). Instead, the central bank points to protecting its own balance sheet but also justified these measures with reference to the secondary objectives; to “support the green transition of the economy in line with the EU’s climate neutrality objectives” (ECB, 2022). To this end, the ECB announced five types of measures. First, effective already, corporate bond holdings have been reviewed and new investments will from now on be tilted “towards issuers with better climate performance through the reinvestment of the sizeable redemptions expected over the coming years”. However, as the ECB also notes, the actual volume of purchases would be determined “solely by monetary policy considerations and their role in achieving the ECB’s inflation target”. Second, the ECB collateral framework would similarly be redesigned to limit the eligibility of debt issued by firms “with a high carbon footprint” (expected to be effective in 2024). Third, the ECB will require all eligible collateral to comply with the EU’s Corporate Sustainability Reporting Directive (CSRD) (expected from 2026 onwards). For asset-backed securities and covered bonds, the ECB will take measures to harmonise disclosures and push for more stringent policies. Fourth, and potentially the most impactful, the ECB will “urge rating agencies to be more transparent about how they incorporate climate risks into their ratings and to be more ambitious in their disclosure requirements on climate risks”. Finally, the in-house credit assessment facilities of the national central banks will become subject to common minimum standards for including climate-related risk in their ratings by the end of 2024.

Like the conditions that the Banque de France attached to its credit ceilings, these measures apply to instruments whose primary purpose is not allocative but geared towards monetary stability (ECB, 2021). The ECB does not implement corporate purchases or engage in refinancing operations primarily to support EU climate policy, but rather to achieve its broader macroeconomic policy objectives. However, in doing so, its new strategy sees it make design choices geared towards supporting market practices that fit the EU’s broader climate agenda and avoid unduly incentivizing investments that conflict with it. Once again, it takes ideological rigour to find much that is substantively objectionable about this return to “Keynesian” practices (cf Monnet, 2018, p. 13). But what about legitimacy when the policies, in contrast to the moderate interventionism of the 70s, are executed by technocrats?

## 3 A Credit Council Today

The ECB’s legal framework always contained ample scope to move towards moderate interventionism. However, reflecting the vision of a starkly independent central bank, the accountability provisions it is strictly bound by remain terse.

The European Central Bank is for now conspicuously lacking the broader institutional context in which the Banque de France’s credit policy was embedded (Monnet, 2021a, 2022). In the 18 months of its strategy review, the central bank itself set out a new interpretation of the mandate and defined the criteria against which future interventions would be measured. In this way, the ECB’s review remained insulated from societal processes of deliberation and decision-making. This has meant, as Monnet has also pointed out, that to date the ECB’s invocation of its secondary mandate remains very selective. The central bank justifies its choices by selectively invoking some of the many objectives from Article 3 TEU it is meant to support, but the central bank does not provide an account of alternative policy options and reasons to choose one approach over another.

The ECB’s old basis of democratic legitimacy rested on its task being narrow; using one instrument to achieve a well-defined objective price stability (Lastra, 2015; Magnette, 2000; Scheller, 2006). But while this is now a thing of the past, the central bank still holds on to a strict reading of its independence (Amtenbrink, 2019; Borger, 2020; Beukers et al., 2022; Högenauer & Howarth, 2019; Markakis, 2020; Tuori, 2019; van ’t Klooster & Grünewald, 2022). The crucial provision sets out the ECB’s unprecedented independence has two aspects. First, and crucial for the legality of the ECC, central bankers are not allowed to take “instructions”:

When exercising the powers and carrying out the tasks and duties conferred upon them by the Treaties and the Statute of the ESCB and of the ECB, neither the European Central Bank, nor a national central bank, nor any member of their decision-making bodies shall seek or take instructions from Union institutions, bodies, offices or agencies, from any government of a Member State or from any other body. (TFEU 130)

At the same time, the European Parliament and other institutions are not allowed to interfere with ECB decision-making:

The Union institutions, bodies, offices or agencies and the governments of the Member States undertake to respect this principle and not to seek to influence the members of the decision-making bodies of the European Central Bank or of the national central banks in the performance of their tasks. (TFEU 130)

I agree with Monnet (and many others) that strict independence is hard to reconcile with the ECB’s new role. Unlike its simple 1998 and 2003 strategies, the ECB today faces complex distributive choices in the implementation of monetary policy. This implies that we should look to scope available within the context of this provision for more inter-institutional coordination. However, the devil is in the details and it is easier to assert than ensure that the ECC will have a meaningful role while the ECB at the same time retains “full operational autonomy and policy independence” (Monnet 2022, p. 20).

Read in the context of the broader treaty, it quickly becomes clear that the provisions on central bank independence leave ample scope for inter-institutional coordination aimed at achieving the ECB’s monetary policy objectives (Ioannidis et al., 2021; Van ’t Klooster & de Boer, 2022). For example, the European Parliament, as the ECB’s primary accountability forum, has the right to hear the ECB’s President and to debate its annual report. These types of provisions would not have any purpose if they did not in any way shape how the ECB pursues its role. Accordingly, as Advocate-General Jacobs of the Court of Justice of the EU stated in the OLAF case, the ECB’s independence

does not imply […] a complete absence of cooperation with the institutions and bodies of the Community. The Treaty prohibits only influence which is liable to undermine the ability of the ECB to carry out its tasks effectively with a view to price stability, and which must therefore be regarded as undue.3Opinion AG Jacobs in Case C-11/00, *Commission of the European Communities v European Central Bank*, ECLI:EU:C:2002:556, (hereafter: Opinion AG Jacobs *OLAF*), par. 155, also par. 156–158.

How then should the relationship between the ECB and the ECC be cast? Any coordination that limits the ECB’s policy autonomy concerning the price stability objective would be hard to reconcile with the treaties. That concern is less pressing when the ECB acts on its secondary objectives. Here, the central bank’s role is explicitly supportive of EU policy, creating scope for guidance by the ECC, suitably empowered by other EU institutions to set out what and how to provide support. Even concerning the secondary objective, the ECC should not be conceived as an institution from which the ECB receives (binding) instructions. Instead, the source of the legal requirement to support the EU’s broader economic policies can only be the secondary mandate itself. The ECC provides the ECB with evidence concerning how to interpret its vague provisions. It is this role in prioritizing for which officials of the legal department of the ECB itself also suggested that the European Parliament could take a more prominent role (Ioannidis et al., 2021) and which the EP has increasingly taken up (EP, 2021).

Contributing to this work of the EP, and building on Monnet, I think there are at least three permissible ways for the Credit Council to exercise influence over the ECB. First, the ECC should take the lead in a process of inter-institutional coordination for designing a new ECB accountability framework as well as determining what secondary objectives the central bank should be supporting. The provisions as drafted are broad and defy any straightforward interpretation as the competencies for making economic policies are distributed amongst EU and member state decision-making bodies. Ideally, a shared vision of what constitutes the priorities of economic policy could emerge from such a dialogue, providing the ECB with crucial evidence for the design of its operations.

Second, the ECC should help the European Parliament, as well as the European Commission and the Council to draft effective legislation that can be incorporated into ECB policies. The unique volume of legislation passed by the European legislator creates a need to set priorities. A clear reference to the ECB’s secondary mandate could be incorporated into EU laws that have relevant implications for the design of monetary policy. For example, regulations like the CSRD, which the ECB already relied on in its July 2022 greening measures, and the EU taxonomy could be flagged as relevant to the interpretation of Article 127(1) in their preamble. Now that much progress has been made on capital markets, greening the ECB’s TLTRO programme should be the next priority.

Finally, the ECC could have a leading role in overseeing the constitutional embedding of the ECB itself. Central bank independence cannot mean a permanent and irrevocable delegation of tasks in the face of changing circumstances (Downey, 2021). The treaties provide for an overlooked set of tools for revising key passages in the ECB mandate. Alongside work on a new toolbox (Statutes Article 20) and use of the simplified amendment procedure (Statutes Article 40), highlighted by Monnet, Article 125.2 TFEU also foresees in a procedure to put forward an interpretation of the monetary financing prohibition and the privileged access provision. Over time, work along these lines could evolve into a more definite view of what constitutional amendments would be desirable in the event of a constitutional convention.

## 4 Conclusion

I examined Eric Monnet’s proposal for a European Credit Council by situating it in the history laid out in his 2018 study *Controlling Credit*. I drew from the 1970s a tradition that I described as “moderate interventionism” and existed alongside market-based operational frameworks throughout the postwar era. I showed that the drafters of the ECB statutes left ample scope for such interventionism, paving the way for a transformation of the ECB that culminated in the 2021 monetary policy strategy. Against this historical background, and drawing on earlier work, I suggested that there is ample scope within the treaties for an ECC.

The most important legal constraint on the ECC is that it cannot make legally binding instructions. At least within the existing legal framework, the ECB will always retain the sole authority to decide how to pursue price stability. In this regard, the ECC offers a modest solution, within the context of the treaties, to glaring gaps in ECB accountability practices. These gaps reflect an antiquated treaty, which does not come with a parliamentary body that can revise the most important treaty provisions. This, in turn, is a consequence of the Euro area’s decentralized political system and the absence of a strong European public sphere. Can the ECC help build the Euro-polity needed for a genuinely democratic central bank?
